# The effects of HIV and oncogenic human papillomavirus on the tumor immune microenvironment of penile squamous cell carcinoma

**DOI:** 10.1371/journal.pone.0300729

**Published:** 2024-05-01

**Authors:** Chibamba Mumba, Zoran Muhimbe, Victor Mapulanga, Musonda Kawimbe, Keagan Mutale, Anglin Hamasuku, Jane Musumali, Nicholas K. Mwale, Owen Ngalamika

**Affiliations:** 1 Department of Pathology and Microbiology, School of Medicine, University of Zambia, Lusaka, Zambia; 2 Department of Surgery, School of Medicine, University of Zambia, Lusaka, Zambia; 3 HHV8 Research Molecular Virology Laboratory, University Teaching Hospital, Lusaka, Zambia; 4 Department of Physiological Sciences, School of Medicine, University of Zambia, Lusaka, Zambia; 5 Dermatology and Venerology Division, School of Medicine, University of Zambia, Lusaka, Zambia; University of Health and Allied Sciences, GHANA

## Abstract

Penile squamous cell carcinoma (PSCC) occurs more frequently in some developing countries compared to developed countries. Infection with HIV and/or high-risk human papillomavirus (hrHPV) are risk factors for penile cancer development. The tumor microenvironment of PSCC may predict prognosis and may inform on the best targets for immunotherapy. We evaluated the immune microenvironment of penile tumors histologically, and determined whether and/or how HIV and/or hrHPV infections affect this tumor microenvironment. We conducted a prospective analytical cross-sectional study in which penile cancer tumors from 35 patients presenting at the University Teaching Hospital in Lusaka, Zambia were histologically staged and assessed for presence of tumor infiltrating immune cells and expression of immune checkpoints. Immunohistochemistry was used to evaluate immune checkpoints and infiltrating immune cells, while multiplex real-time polymerase chain reaction was used for hrHPV genotyping. The median age of all participants was 55 years. About 24% had advanced histological stage, 83% were HIV^+^, and 63% had hrHPV detected in their tumors using multiplex real-time polymerase chain reaction. PDL1 expression was significantly higher in HIV^-^ participants than HIV^+^ participants (p = 0.02). Tumors with multiple hrHPV infections had a significantly higher number of cells expressing TIM3 than those with one hrHPV (p = 0.04). High grade tumors had a significantly higher infiltrate of FoxP3^+^ cells (p = 0.02), CD68^+^ cells (p = 0.01), CD163^+^ cells (p = 0.01), LAG3^+^ cells (p = 0.01), PD1^+^ cells (p = 0.01) and TIM3^+^ cells (p = 0.03) when compared with low grade tumours. There was significant moderate to strong positive correlation of cells expressing PD1 and LAG3 (⍴ = 0.69; p = 0.0001), PD1 and TIM3 (⍴ = 0.49; p = 0.017) and TIM3 and LAG3 PDL1 (⍴ = 0.61; p = 0.001). In conclusion, the tumor microenvironment of penile squamous cell carcinoma seems to be affected by both HIV and HPV infections. TIM3 appears to be a potential therapeutic target in PSCC patients with hrHPV infections.

## Introduction

Penile squamous cell carcinoma (PSCC) is the most common malignancy of the penis, and although considered to be rare in the developed countries, occurs more frequently in some developing countries [1–3]. In most Western, Middle East, and some North African countries, the incidence of PSCC is less than l case per 100,000 persons, whereas the incidence is much higher (3 to 7 cases per 100,000 persons) in some South American, Asian, and Sub-Saharan Africa (SSA) countries [3–5]. Infection with human papillomavirus (HPV) is among the risk factors associated with PSCC development [6]. Thus, PSCC is now classified as HPV-associated or HPV-independent, due to the distinct pathogenesis pathways of the two types of tumors [7].

HPV-associated PSCCs are more common in SSA countries where the incidence of the malignancy is high. Patients in these countries present at a much younger age than patients in first-world countries where the incidence is much lower [8]. In addition, most patients in SSA present with advanced disease, and a majority are infected with the Human Immunodeficiency Virus (HIV) [9]. HIV infection has been observed to shorten the progression time from in-situ carcinoma to invasive carcinoma, with an increased risk of death from the cancer [10]. These factors are thought to contribute to the observed poor prognosis for PSCC patients in both developed and developing countries [11, 12].

The PSCC tumor microenvironment may be a predictor of prognosis and susceptibility to immunotherapy. HPV-positive PSCCs have been observed to have a higher stromal CD8^+^ T cell infiltrate when compared to HPV-negative tumors [13]. In addition, a high density of CD68^+^ tumor macrophages have been associated with a better median cancer-specific survival, median overall survival, and lower risk of recurrence [14]. In a recent meta-analysis, it was observed that over-expression of the immune checkpoint PDL1 in PSCCs is associated with worse survival outcomes [15]. In addition, HPV-negative tumors have been observed to have higher PDL1 expression than HPV-positive tumors [16], with excellent response to PD-1 inhibitors in patients who have PD-L1 overexpression in the tumors [17].

Despite HIV infection associating with an increased risk of penile cancer, its influence on the PSCC tumor microenvironment is currently not well understood. In other malignancies such as anal squamous cell carcinoma, it has been suggested that HIV-induced chronic inflammation may upregulate PD-1 expression resulting in CD8 T cell exhaustion, and failure of the T cell to kill the tumor [18]. In this study, we sought to evaluate the immune microenvironment of PSCC histologically, and determine whether and how HIV and/or HPV infections may affect this environment.

## Methods

### Study design and participants

We carried out a prospective analytical cross-sectional study of penile squamous cell carcinomas from 28^th^ November 2022 to 2^nd^ October 2023. The study participants were recruited from the Urology Clinic of the University Teaching Hospital in Lusaka, Zambia upon obtaining written informed consent. The consenting participants were consecutively enrolled in to the study after histological confirmation of the PSCC. At time of enrollment, a questionnaire was administered to collect sociodemographic and clinical information including age, smoking status, and HIV infection status. The penile tumors were obtained in theatre upon partial or total penectomy. The fresh PSCC tumors were then sent to the histopathology laboratory for grading, staging, and sampling for other subsequent investigations including immunohistochemistry (IHC) and HPV genotyping. Ethical approval to conduct this study was obtained from the University of Zambia Biomedical Research Ethics Committee (Ref. No.: 3233–2022) and the Zambia National Health research Authority (Ref No.: NHRA0000010/31/10/2022).

### HIV viral load and CD4 counts

At the time of recruitment, venous whole blood was collected for HIV viral load detection and CD4 counting. HIV-1 plasma viral load was measured on the Hologic Panther (Hologic) using the Aptima HIV-1 Quant Dx Assay kit (Hologic), according to the manufacturer’s protocol. For the purpose of analysis, HIV viral loads below the detection limit (<30copies/ml) were recorded and analyzed as zero. CD4 counts were determined using the BD TriTest kit (BD Biosciences) on a BD FACSCalibur instrument (BD Biosciences), according to the manufacturer’s protocol.

### HPV detection and genotyping

DNA was extracted from the tumor using a commercial nucleic acid extraction kit (QIAamp^R^DNA Mini Kit). Detection of HPV genotypes was done using multiplex real-time PCR on a CFX96^TM^ Real-time PCR detection system (BIO-RAD), using the Anyplex^TM^ II HPV28 detection kit according to the manufacturer’s instructions. The kit and PCR platform allow for detection of 19 high-risk (16, 18, 26, 31, 33, 35, 39, 45, 51, 52, 53, 56, 58, 59, 66, 68, 69, 73, and 82) and 9 low-risk (6, 11, 40, 42, 43, 44, 54, 61, and 70) HPV types.

### Immunohistochemistry

We performed immunohistochemical staining on formalin-fixed paraffin-embedded penile tumors. We stained for CD3, CD4, CD8, FoxP3, CD168, CD63, PD-1, PDL-1, TIM-3, LAG3, p16, p53, MLH1, MSH2, MSH6, and PMS2. Briefly, 4 micrometer sections were mounted on X-tra adhesive slides (LeicaBiosystems). Positive controls for each marker were incorporated in each run, and were either a palatine tonsil, lymph node, or a tissue known to be positive for a particular marker. Negative controls included a tissue known to be negative for a particular marker, or no addition of the primary antibody. The sample and control tissues were baked for 1–2 hours at 60°C. Antigen retrieval was then performed using the semi-automated PT Link (Agilent), according to the manufacturer’s product instructions and guidelines. The PT link allows for deparaffinization, rehydration, and antigen retrieval combined in a 3-in-1 specimen preparation procedure [19]. After some washing and blocking steps, the primary antibody was applied and samples left for an hour, then washed in wash buffer. The samples were then incubated with post primary linker (Novolink polymer detection; Leica Biosystems) for 30minutes, and then peroxidase activity was developed using Diaminobenzidine working solution. Background staining was subsequently done with hematoxylin.

Immunohistochemically stained slides were assessed by two independent reviewers. The density of tumor infiltrating immune cells (TIIC) was assessed microscopically at high power magnification (x400) in five different fields that were representative of the tumor. Counting was done in the intra-tumoral compartment and expressed as count in a high-power field. PD-L1 expression was assessed on the membrane of tumour cells and tumour infiltrating immune cells (macrophages and lymphocytes) (S1A Fig). This assessment was made at a microscopic magnification of x20. The number of viable tumor cells was also assessed at the same magnification. Using the two assessments, a combined proportionate score (CPS) was then calculated. CTLA-4 was scored using the CPS as described above (S1B Fig). LAG3 expression was assessed as staining of cytoplasmic and or membrane staining. PD-1 and TIM3 expression was determined by counting the number of lymphocytes expressing this marker in 5 representative high-power fields of the tumor (S1C and S1D Fig respectively).

### Data analysis

Summary statistics were used for baseline characteristics. Chi-square test was used to determine associations between two dichotomous variables. Wilcoxon rank-sum test was used to determine any differences in continuous variables between dichotomous groups. Spearman’s rank correlation was used to determine any correlations between continuous variables. Comparisons were made by histological stage, HIV status, hrHPV status, HIV/hrHPV co-infection status, presence of multiple hrHPVs, tumor grade, and primary vs. metastatic tumors. P values <0.05 were considered statistically significant. STATA version 17 was used to perform all statistical analyses, and Graph pad prism version 9 was used to generate the figures.

## Results

### Baseline characteristics of study participants

We enrolled 35 participants in the study. Participants had a median age of 55 years, about half had a history of smoking, and 82.9% were HIV positive. Table 1 shows the rest of the baseline characteristics of the study participants. Among the HPV-positive tumors, the most common genotype among all the tumors was 16 (55%), followed by 35 (50%), then 18, 33, and 53 (18% each). Expression of the mismatch repair proteins (MLH1, MSH2, MSH6, and PMS2) were normal in all the tumors, and hence not included in any of the analyses.

**Table 1:** Baseline characteristics of study participants.

Median Age (Years)	55[47–62]
Smoking	18/35 (51.40%)
Duration of lesion (months)	8[6–12]
Advanced stage disease	8/33 (24.2%)
HIV positive	29/35 (82.9%)
HIV Viral Load (copies/ml)	0[0–0]
CD4 Count (cells/μl)	466.5[328.5–695]
HPV positive	22/35 (62.9%)
HIV/hrHPV Co-infection	19/35 (54.3%)

### Tumor immune microenvironment of early vs advanced stage disease on histology

The TNM staging system was used to histologically stage the participants, and those with lymph node metastasis were considered as advanced stage. Tumors were categorized as early stage (stages I and II) and advanced stage (stages III and IV). Patients with advanced stage disease had a lower median age compared to those with early-stage disease, though the difference was only marginally significant (Table 2). There were no differences in numbers of infiltrating immune cells and expression of immune checkpoint molecules between early-stage tumors compared to advanced stage tumors.

**Table 2:** Factors associated with advanced stage disease.

	Early Stage	Advanced Stage	p Value
	(N = 25)	(N = 8)	
Median Age (Years)	58[54–63]	50.5[43–55.5]	0.065
Smoking	48%	62.5%	0.48
Duration of lesion (months)	8[6–12]	9[5–30]	0.78
HIV positive	88%	75%	0.37
HIV viral load (copies/ml)	0[0–0]	0[0–0]	0.32
CD4 count (cells/μl)	497[343–696]	521[273–729]	0.98
High risk HPV in tumor	56%	87.5%	0.11
p16 Positive	21/24 (87.5%)	87.5%	1.00
Abnormal p53 expression	3/23 (13%)	12.5%	0.97
CD3^+^ cells	39[17–61]	52.5[35–111]	0.39
CD8^+^ cells	26[10–42]	41.5[28.5–58.5]	0.23
CD103^+^ cells	22[11–77]	49[27.5–65]	0.38
FOXP3^+^ cells	10[5–13]	16[7–22]	0.18
CD68^+^ cells	17.5[11–32]	20.5[14.5–32]	0.70
CD163^+^ cells	23[16.5–40]	34[20–51]	0.32
LAG3^+^ cells	17[8–30]	15.5[9–31.5]	0.97
PD1^+^ cells	13.5[4–37]	10.5[3–26]	0.73
TIM3^+^ cells	20[5.5–36.5]	21[3.5–35.5]	0.85
PDL1^+^ cells	20[0–55]	8.5[4–21.5]	0.52
CTLA^+^ cells	0.8[0–2.3]	0.73[0.1–4.2]	0.62

### Comparison of the penile tumor microenvironments by HPV status

**Fig 1:**
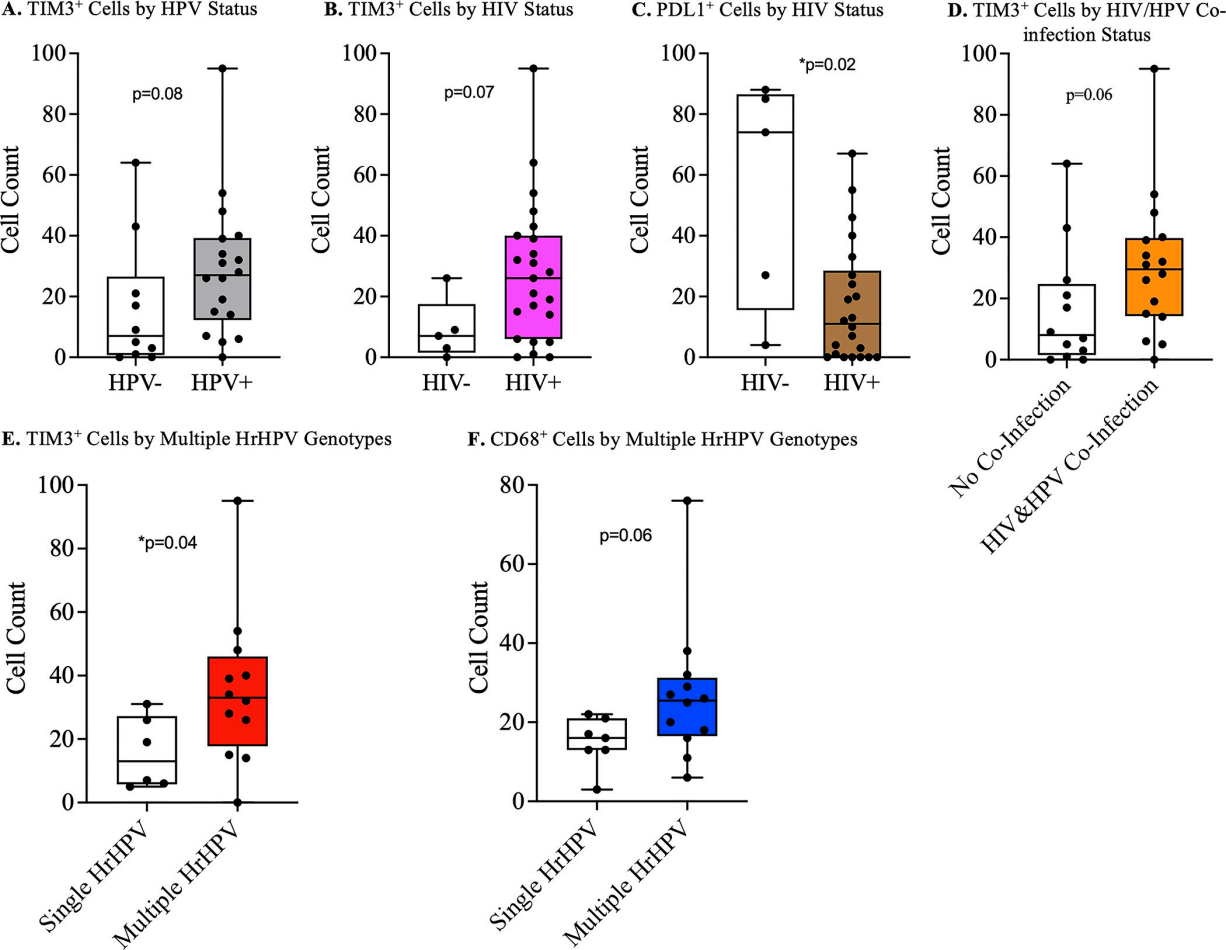
Penile cancer tumor immune microenvironment by HPV and HIV status. A) Higher but statistically insignificant number of lymphocytes expressing TIM3 in hrHPV-associated than hrHPV-independent tumors. B) Statistically insignificant higher numbers of lymphocytes expressing TIM3 in penile tumors of HIV positive compared to HIV negative individuals. C) Significantly higher PDL1 expression in penile tumors from HIV negative compared to HIV positive individuals. D) HrHPV-associated penile tumors in HIV infected individuals had a higher number of lymphocytes expressing TIM3. E) Penile tumors with multiple hrHPV genotypes had significantly higher number of lymphocytes expressing TIM3 than tumors with a single hrHPV. F) Statistically insignificant higher number of macrophages in tumors with multiple hrHPV genotypes compared to tumors with a single hrHPV genotype.

More HPV-associated tumors had an advanced stage disease compared to HPV-negative tumors, though this difference was not statistically significant (S1 Table in S1 File). There was no significant difference in the immune cell infiltrates between HPV-associated penile squamous cell carcinomas and non-HPV associated carcinomas. However, with regards immune checkpoints, TIM3 expression was higher among HPV-associated tumors than HPV negative tumors. However, this difference was not statistically significant (p = 0.08) (Fig 1A).

### Comparison of the penile tumor microenvironment by HIV status

HIV positive participants had a significantly higher proportion of tumors expressing p16 (p = 0.001), and a significantly lower proportion of tumors with abnormal p53 expression compared to tumors from HIV negative participants (S2 Table in S1 File). There was no difference in numbers of infiltrating immune cells between the HIV negative and HIV positive participants, but differences were noted in the expression of immune checkpoints. The number of immune cells expressing TIM3 was higher in HIV positive participants when compared with HIV negative participants, though the difference was not statistically significant (p = 0.07) (Fig 1B). PDL1 expression was significantly higher in HIV negative participants compared to HIV positive participants (p = 0.02) (Fig 1C).

### HIV and hrHPV co-infection and the penile squamous cell carcinoma microenvironment

There was no difference in p16 and p53 expressions, including tumor infiltration of immune cells between participants with HPV/HIV co-infection and those without (S3 Table in S1 File). On the other hand, there was a statistically insignificant higher number of immune cells expressing TIM3 in participants with co-infection (p = 0.06) (Fig 1D). A comparison of tumors from individuals with only HIV to those with HIV/hrHPV co-infection was non-revealing (S4 Table in S1 File). However, there was a statistically insignificant higher proportion of individuals with advanced stage disease among the co-infected group than the HIV-only group (p = 0.06). A comparison of co-infection with hrHPV-only was not done due to very low number of participants in the hrHPV-only group.

### Effect of multiple high-risk human papilloma virus (HrHPV) genotypes on penile tumor immune microenvironment

Immune cells expressing the immune checkpoint TIM3 were significantly numerous in tumors that had multiple HrHPV genotypes when compared with those that had a single HrHPV genotype (p = 0.04) (Fig 1E). The other immune checkpoints we investigated were not significantly different between the two groups of tumors (S5 Table in S1 File). Tumors with multiple high-risk HPVs generally had a higher number of infiltrating immune cells when compared with those with a single high-risk HPV (S5 Table in S1 File). However, these differences were not statistically significant (Fig 1F).

### Characteristics of the tumor immune microenvironment of low versus high grade penile squamous cell carcinomas

**Fig 2:**
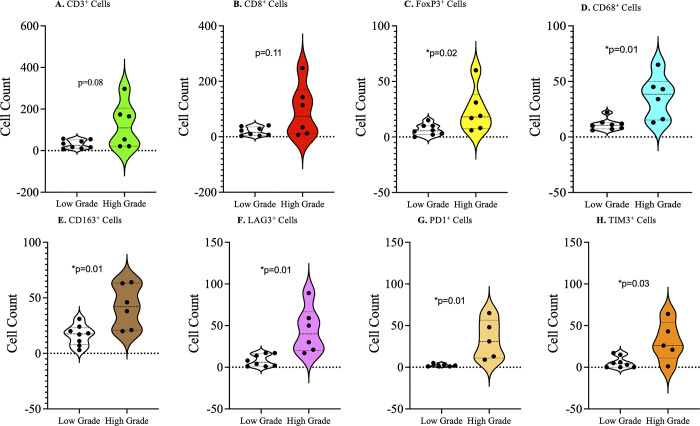
Tumor infiltrating immune cells and immune checkpoint expression by histological grade. A) Higher number of infiltrating T cells in high-grade lesions, with no statistical significance. B) Statistically insignificant higher number of infiltrating CD8^+^ cells in high-grade lesions compared to low-grade lesions. Statistically significant higher numbers of regulatory T cells (C), macrophages (D), and tumor associated macrophages (E) in high-grade penile tumors compared to low-grade tumors. Statistically significant higher number of lymphocytes expressing LAG3 (F), PD1 (G), and TIM3 (H) in high-grade penile cancer tumors compare to low-grade tumors.

Tumors were grouped into 3 grades (grade 1: well-differentiated; grade 2: moderately differentiated; and grade 3: poorly differentiated). When grade 1 and grade 3 tumors were compared, high grade tumors had a higher infiltrate of immune cells and a higher expression of immune checkpoints compared to low grade tumors (Fig 2). The infiltrate of CD3 positive cells was higher but not statistically significant in high grade penile squamous cell carcinomas (p = 0.08) (Fig 2A), while the number of cells expressing FOXP3, CD68 and CD163 were significantly higher in the high-grade tumors when compared with the low-grade tumors, Fig 2C–2E respectively. The number of immune cells expressing the immune checkpoints LAG3, PD1, and TIM3 were significantly more numerous in grade 3 than grade 1 penile squamous cell carcinomas (Fig 2F–2H respectively).

### Correlation of immune checkpoint molecule expression

**Fig 3:**
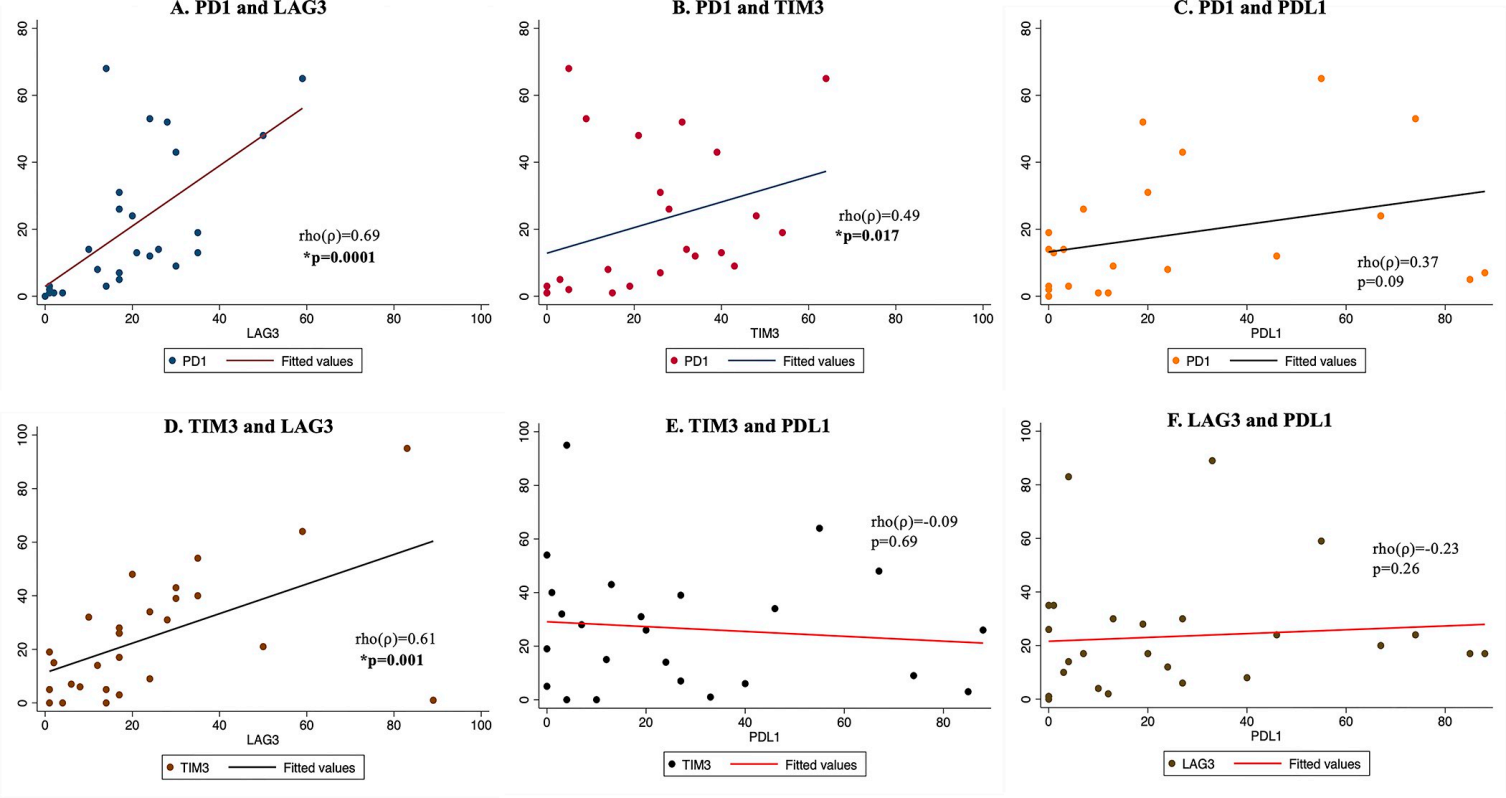
Co-expression of immune checkpoint molecules in penile cancer tumors. A) Strong, statistically significant, positive correlation in PD1 and LAG3 expression on immune cells in penile tumors. B) Moderate, statistically significant, positive correlation in PD1 and TIM3 expression on immune cells in penile tumors. C) Statistically insignificant correlation in PD1 and PDL1 expression. D) Strong, statistically significant, positive correlation in TIM3 and LAG3 expression on immune cells in penile tumors. No correlation in expression of TIM3 and PDL1 (E), and LAG3 and PDL1 (F), in penile tumors.

Correlation between immune checkpoints was evaluated using Pearson’s correlation coefficient. Expression of PD1 and LAG3, and TIM3 and LAG3 were positively significantly correlated with the former being more strongly correlated (Fig 3A and 3D). There was a significant moderate positive correlation between PD1 and TIM3 in the tumors (p = 0.017) (Fig 3B). There was a weak, positive, statistically insignificant correlation between PD1 and PDL1expression (Fig 3C). There was no correlation in expression of TIM3 and PDL1 (Fig 3E) and between LAG3 and PDL1 (Fig 3F) in the penile tumors.

### Primary tumor vs metastasized tumor

**Fig 4:**
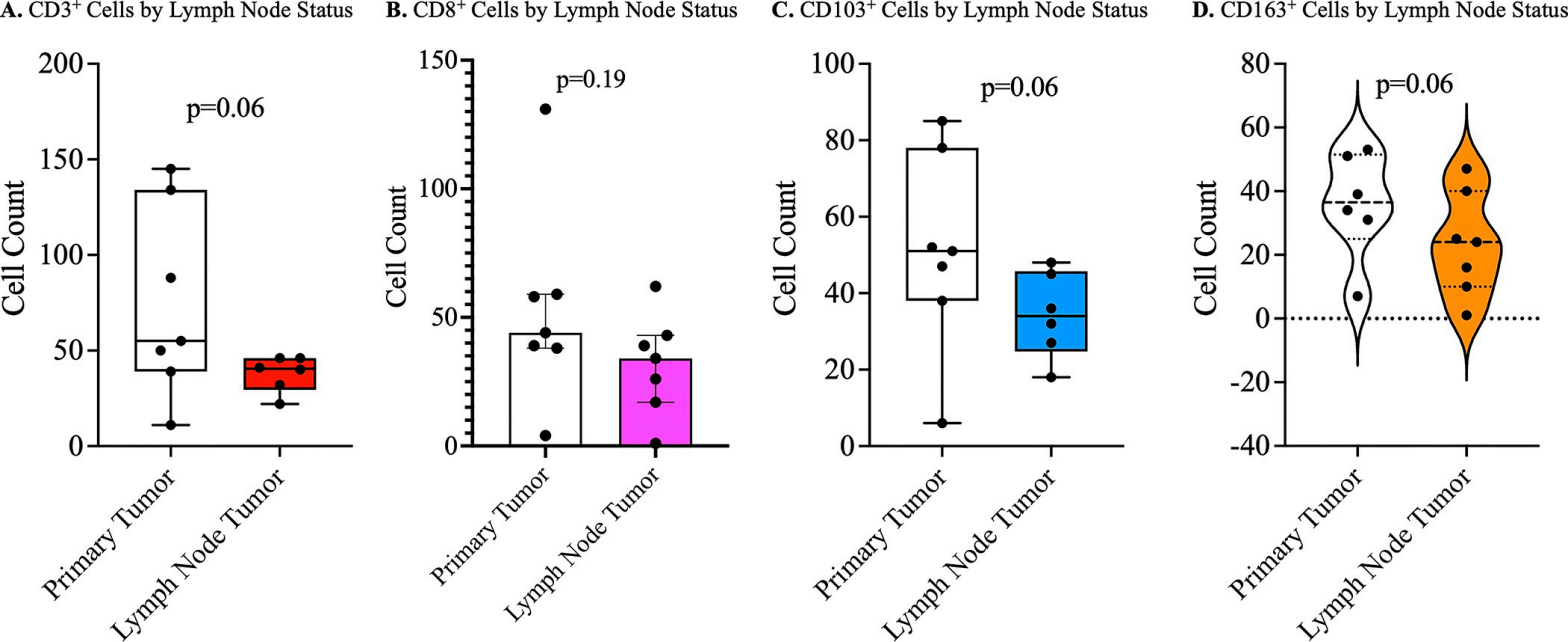
Tumor microenvironment of primary versus metastatic penile cancer lesions. Statistically insignificant lower infiltration of CD3^+^ (A), CD103^+^ (C), and CD163^+^ (D) cells in metastatic penile tumors compared to the primary tumors. No significant difference in the number of CD8^+^ T cells (B) in metastatic tumor compared to primary tumor.

The primary tumor was compared with the lymph node metastasis with regards to immune cell infiltration and immune checkpoint expression. There was no difference in the expression of immune checkpoint molecules on immune cells in the primary compared to metastatic penile squamous cell carcinomas in the same patient. There were higher immune cell infiltrations of CD3 (lymphocytes), CD103 (resident memory lymphocytes), and CD163 (M2 macrophages) positive cells in the primary compared to metastatic tumors (Fig 4). However, these differences were only marginally significant.

## Discussion

Both HIV and hrHPV infections are associated with an increased risk of penile cancer development [6, 10, 20, 21]. These infections may also have an effect on the penile cancer tumor microenvironment, which may affect prognosis and may require a different approach in use of immune therapy. The main objective of this study was to determine how both HIV and hrHPV infections affect the tumor immune microenvironment of penile cancer. About 80% of our study population were HIV positive, and about 63% had hrHPV in the tumor. This observation is different from developed countries where approximately 30% of penile tumors had hrHPV detected [22]. We observed significant differences in expression of immune checkpoint molecules and infiltrating immune cells in penile tumors by HIV status and hrHPV status.

Some previous studies and case reports have reported that HIV infection is associated with a higher risk of progression from in-situ to invasive penile cancer [10, 23]. In our study, there was no significant difference in the proportion of HIV positive individuals between those with advanced histological stage and those with early-stage disease. On the other hand, we observed a significantly higher proportion of p16-expressing penile tumors among HIV positive individuals, and a significantly lower proportion of tumors with abnormal p53 expression among the HIV positive. This was in line with another observation we made in this study that a higher proportion of penile tumors from HIV positive participants had hrHPV detected compared to tumors from HIV negative individuals. The tumor suppressor p16 is a surrogate marker for HPV-associated penile cancer [24], and hence the higher detection of both p16 and HPV among HIV positive tumors. HPV-associated penile cancer is mostly induced by HPV oncoproteins, while HPV-independent penile cancer is induced by mutations in tumor suppressors including p53 and pRb [25]. Our findings suggest that a majority of HIV positive PSCC patients in our population have HPV-associated disease compared to HIV negative patients who seem to have more of HPV-independent disease.

We observed no differences in infiltration of immune cells in penile tumors from HIV positive compared to tumors from HIV negative individuals. However, there were some significant differences in immune checkpoint expression by HIV status. PDL1 expression was significantly higher in penile tumors from HIV negative compared to HIV positive individuals. PD-L1 expression has been observed to be present in penile tumors, with higher expression associated with poor survival [15]. Our findings suggest a higher expression in penile cancer patients who are HIV negative, and may be more useful as an immunotherapeutic target for that population. PD-L1 expression was also higher, although statistically insignificant, in HIV/hrHPV co-infected individuals compared to others without the co-infection.

Despite hrHPV being an important risk factor for development of penile cancer, some studies have observed that patients with hrHPV-associated penile cancer have a better overall and disease-free survival than those with hrHPV-independent cancer [26]. In a study by Scheiner *et al*., no association was observed between hrHPV presence in the tumor and disease stage or metastasis [27]. This is similar to our study where we observed no association between HPV status and histological stage of the tumor. Expression of the immune checkpoint TIM3 was higher in penile tumors with hrHPV compared to tumors without hrHPV. Also, TIM3 expression was significantly higher in tumors with multiple hrHPV compared to tumors with only one hrHPV. TIM3 expression in relation to HPV expression has not been studied in penile cancer. However, it has been observed to be highly expressed on lymphocytes in HPV-associated cervical cancer tumors [28]. TIM3 expression on lymphocytes in cervical cancer tumors has been associated with cancer progression [29, 30]. TIM3 may therefore be a potential immunotherapy target for penile cancer, especially HPV-associated penile cancer, similar to other squamous cell carcinomas such as HPV-associated cervical cancer.

Monotherapy with immune checkpoint inhibitors is often ineffective, and hence combination therapy has proven to be more effective for most cancers [31]. It has been previously observed that tumors resistant PD-1/PD-L1 therapy have an upregulation of TIM3-expressing tumor infiltrating lymphocytes [32]. Also, it has been previously observed that TIM3^+^ tumor infiltrating lymphocytes co-express PD-1, make up a major fraction of T cells infiltrating tumors, and exhibit the most exhausted phenotype [33]. In this study, we have observed moderate to strong correlation in co-expression of PD-1 and LAG3, PD-1 and TIM3, and TIM3 and LAG3. These could be potential targets for combined immune checkpoint blockade therapy for penile cancer patients with tumor infiltrating lymphocytes expressing any of these molecules.

Cancer grade reflects how different the tumor cells are from the normal cells, and is a sign of rapid tumor growth. In penile cancer, studies have observed that a high histological grade is an independent predictor of mortality [34]. In the current study, we have observed a significantly higher number of cells expressing regulatory T cells (Treg) and tumor-associated macrophage (M2 macrophage) markers in high grade compared to low grade tumors. Infiltration of CD3^+^ and cytotoxic CD8^+^ lymphocytes was higher in the high-grade tumors compared to low grade tumors, but not statistically significant. Furthermore, immune checkpoint molecules including LAG3, PD-1, and TIM3 were significantly higher on tumor infiltrating lymphocytes of high grade compared to low grade tumors. These findings may be due to a higher expression of neoantigens as a result of high mutational burden in high grade tumors, leading to the attraction of more immune cells into the tumors. However, this is coupled with a high expression of immune checkpoint molecules on the immune cells, which prevents killing of the cancer cells. Based on our observations, histological grade of penile cancer may be a more reliable indicator for administration of immune checkpoint inhibitor therapy. This is supported by previous studies that have observed that high-grade tumors of squamous cell carcinoma of the head and neck have a better response to immune therapy than low-grade tumors [35]. In addition, a study by Gregoire *et al*., it was observed that presence of hrHPV was associated with high-grade invasive squamous cell carcinoma of the penis [36]. Based on our findings and that of others, HIV and hrHPV status may also be important in determining the targets for immune checkpoint blockade.

The microenvironment of primary tumor has previously been compared with metastatic tumors with regard to infiltrating immune cells. This is because metastatic tumors acquire changes in their genotypes and phenotypes that may affect the tumor infiltrating immune cells and ultimately response to immune therapy [37]. In studies on primary and metastatic breast cancer, it has been reported that metastatic tumors are more immunologically inert, with reduced numbers of tumor infiltrating lymphocytes [38, 39]. This is similar to our study where we have observed a higher presence of tumor infiltrating lymphocytes in primary tumor compared to metastatic tumors. However, our findings were not significant, possibly due to low number of cases as we only included participants with inguinal lymph node metastases for this analysis. No difference was observed in immune checkpoint expression between the primary and metastatic tumors. This suggests that the low presence of infiltrating lymphocytes may be the limiting factor in treatment of metastatic penile tumors with immune checkpoint inhibitors. However, more studies with a larger sample size need to be conducted in order to have a definitive conclusion. To the best of our knowledge, this is the first study done to compare infiltrating immune cells and immune checkpoint molecules in primary versus metastatic penile tumors.

A major limitation in this study is that we did not assess and compare CD4^+^ T cells in the tumors. Also, we were not able to perform multicolor staining of the cells to determine the actual cells expressing the markers of interest. Our initial calculated sample size was 38 participants, and based on a higher number of advanced stage tumors expressing PDL1. However, sample size was not determined for all the markers due to unavailability of preliminary data. We managed to recruit 35 participants during the study period. The low sample size for some sub-analyses including paired comparisons of primary with metastatic tumors was a limitation. Also, we were not able to perform multivariate analyses to control for potential confounders for some analyses due to low numbers in comparative groups.

## Conclusions

The tumor microenvironment of penile squamous cell carcinoma seems to be influenced by HIV and HPV infections. TIM3 appears to be an important immune checkpoint in penile cancer, as it has a higher expression in penile tumors that have multiple hrHPVs, and in HIV/HPV co-infection. PD-L1 expression is higher in penile tumors from HIV negative patients. A high tumor histological grade is associated with a higher infiltration of tumor infiltrating lymphocytes and a higher expression of immune checkpoint molecules. The tumor microenvironment in penile cancers offers potentially actionable therapeutic options, but careful considerations should be made based on HIV and hrHPV status.

## Supporting information

S1 Fig Some markers stained by immunohistochemistry. A) PD-L1 expression on mostly tumor cell membranes (x200 magnification). B) CTLA-4 expression on cell membranes of a few immune cells (x20 magnification). C) PD-1 staining on cell membranes on lymphocytes. D) TIM3 expression on lymphocytes (x200 magnification).
(TIFF)

S1 File (DOCX)

S1 Dataset (XLS)
