# Supplementary figures and images for: The effects of HIV and oncogenic human papillomavirus on the tumor immune microenvironment of penile squamous cell carcinoma
Source: PLoS One. 2024 May 1;19(5):e0300729. doi: 10.1371/journal.pone.0300729 (PMC11062539; doi:10.1371/journal.pone.0300729)

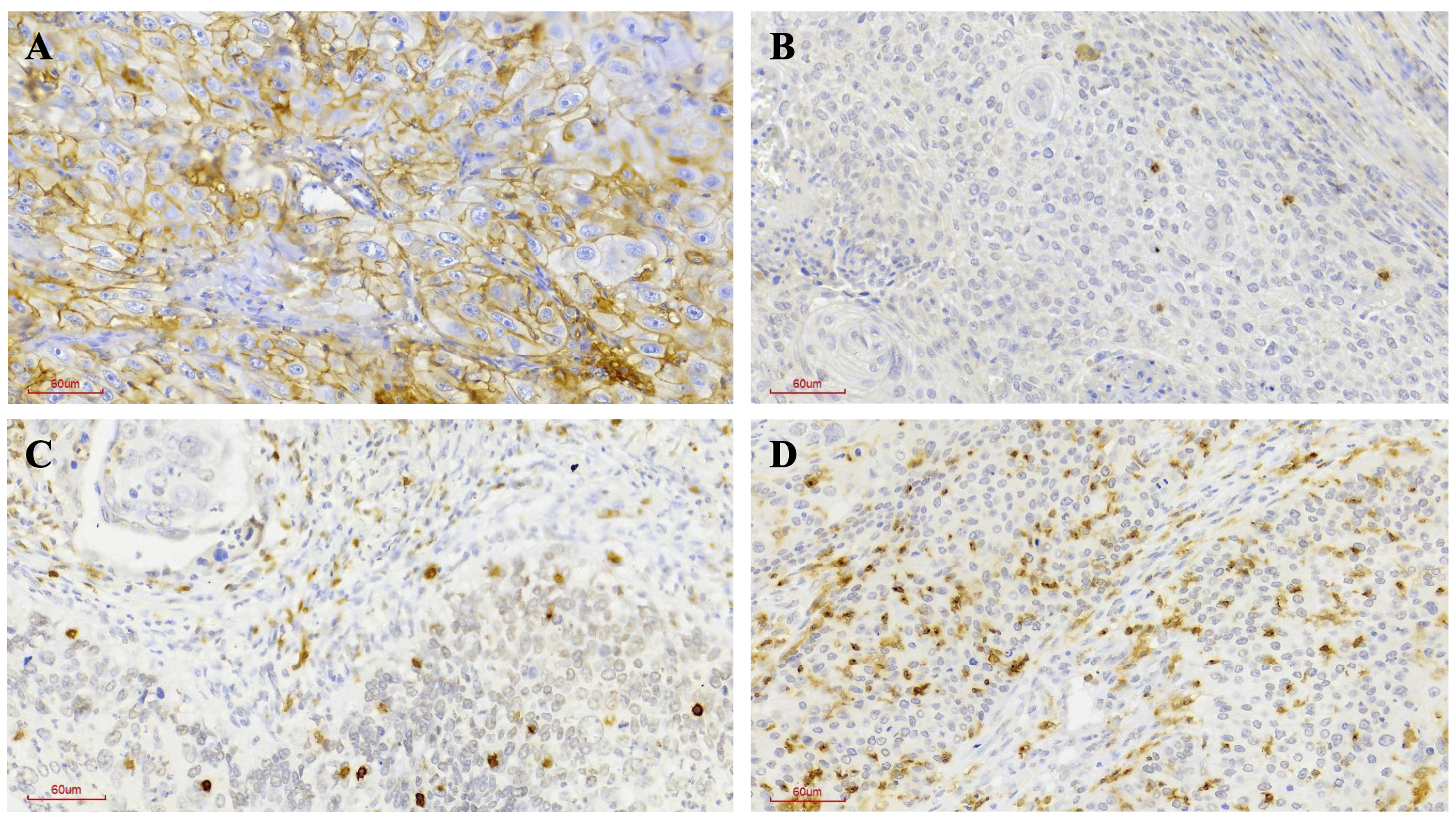

Supplement: S1 Fig — A) PD-L1 expression on mostly tumor cell membranes (x200 magnification). B) CTLA-4 expression on cell membranes of a few immune cells (x20 magnification). C) PD-1 staining on cell membranes on lymphocytes. D) TIM3 expression on lymphocytes (x200 magnification). (TIFF) [file pone.0300729.s001.tiff]
