# Supplementary material for: The effects of HIV and oncogenic human papillomavirus on the tumor immune microenvironment of penile squamous cell carcinoma
Source: PLoS One. 2024 May 1;19(5):e0300729. doi: 10.1371/journal.pone.0300729 (PMC11062539; doi:10.1371/journal.pone.0300729)
Supplement: S1 File — (DOCX) [file pone.0300729.s002.docx]

| **S1 Table.** High-risk HPV and penile squamous cell carcinoma | | | |
| --- | --- | --- | --- |
|  | **HPV Negative (N=13)** | **HPV Positive (N=22)** | **p Value** |
| Median Age (Years) | 58[46-60] | 55[49-63] | 0.94 |
| Smoking | 61.5% | 45.5% | 0.36 |
| Duration of lesion (months) | 8[6-12] | 9[6-24] | 0.99 |
| HIV positive | 76.9% | 86.4% | 0.47 |
| HIV viral load (copies/ml) | 0[0-33] | 0[0-0] | 0.41 |
| CD4 count (cells/µl) | 469[330-651] | 464[289-729] | 1.00 |
| Advanced stage disease | 1/12(8.3%) | 7/21(33.3%) | 0.11 |
| p16 Positive | 9/12(75%) | 19/20(95%) | 0.098 |
| Abnormal p53 expression | 3/12(25%) | 1/19(5%) | 0.11 |
| CD3^+^ cells | 56[19-136.5] | 39[21-54] | 0.37 |
| CD8^+^ cells | 32[8.5-101] | 29[12-44] | 0.68 |
| CD103^+^ cells | 48[11-83] | 28[13-51] | 0.48 |
| FOXP3^+^ cells | 11.5[5.5-18] | 10[5-14] | 0.68 |
| CD68^+^ cells | 13[8-45] | 20[13-27] | 0.99 |
| CD163^+^ cells | 32.5[14.5-46] | 23[17-39] | 0.49 |
| LAG3^+^ cells | 20.5[7.5-40] | 17[8-28] | 0.65 |
| PD1^+^ cells | 7[2-48] | 13.5[7.5-28.5] | 0.48 |
| TIM3^+^ cells | 7[1-21] | 27[14-39] | 0.08 |
| PDL1^+^ cells | 13[0-55] | 15.5[3-27] | 0.93 |
| CTLA^+^ cells | 1.07[0.35-3.05] | 0.5[0-2.3] | 0.33 |

| **S2 Table.** HIV infection and penile squamous cell carcinoma | | | |
| --- | --- | --- | --- |
|  | **HIV Negative**  **(N=6)** | **HIV Positive (N=29)** | **p Value** |
| Median Age (Years) | 52[46-65] | 56[50-61] | 0.76 |
| Smoking | 66.70% | 48.30% | 0.41 |
| Duration of lesion (months) | 6.5[5-8] | 10[6-24] | 0.11 |
| HPV positive | 50% | 65.50% | 0.47 |
| CD4 count (cells/µl) | 778[546-1036] | 458[308-615] | 0.095 |
| Advanced stage disease | 2/5(40%) | 6/28(21.4%) | 0.37 |
| p16 Positive | 2/5(40%) | 26/27(96.3%) | **0.001** |
| Abnormal p53 expression | 3/5(60%) | 1/26(3.8%) | **0.001** |
| CD3^+^ cells | 53[31-55] | 39[19-98] | 0.90 |
| CD8^+^ cells | 34[19-38] | 29[11-54.5] | 1.00 |
| CD103^+^ cells | 22[17-52] | 39[11-77] | 0.91 |
| FOXP3^+^ cells | 12[10-13] | 10[5-17] | 0.67 |
| CD68^+^ cells | 14.5[10-24] | 20.5[12-34] | 0.43 |
| CD163^+^ cells | 31[20-34] | 23.5[17-42] | 0.87 |
| LAG3^+^ cells | 17[14-17] | 18.5[8-30] | 0.57 |
| PD1^+^ cells | 6[4-30] | 13.5[3-31] | 0.67 |
| TIM3^+^ cells | 7[3-9] | 26[6-40] | 0.07 |
| PDL1^+^ cells | 74[27-85] | 11[0-27] | **0.02** |
| CTLA^+^ cells | 0.2[0-3.1] | 0.88[0-2.65] | 0.94 |

| **S3 Table.** HPV&HIV co-infection and penile squamous cell carcinoma | | | |
| --- | --- | --- | --- |
|  | **No Co-infection**  **(N=16)** | **HPV&HIV Co-infection**  **(N=19)** | **p Value** |
| Median Age (Years) | 56.5[47.5-61] | 55[47-63] | 0.85 |
| Smoking | 56.30% | 47.40% | 0.60 |
| Duration of lesion (months) | 8[6-12] | 10[5-24] | 0.71 |
| CD4 count (cells/µl) | 496.5[355-730] | 454.5[273-661] | 0.54 |
| Advanced stage disease | 2/14(14.3%) | 31.60% | 0.25 |
| p16 Positive | 11/14(78.6%) | 17/18(94.4%) | 0.18 |
| Abnormal p53 expression | 3/14(21.4%) | 1/17(5.9%) | 0.20 |
| CD3^+^ cells | 54[21-108] | 39[16-84] | 0.42 |
| CD8^+^ cells | 30[10-88] | 29[12-51] | 0.78 |
| CD103^+^ cells | 33[11-78] | 38[13-51] | 0.67 |
| FOXP3^+^ cells | 11[6-17] | 10[5-14] | 0.60 |
| CD68^+^ cells | 16[10-43] | 20[13-26] | 0.88 |
| CD163^+^ cells | 32.5[18-46] | 23[17-34] | 0.40 |
| LAG3^+^ cells | 17[6-30] | 17[10-28] | 0.96 |
| PD1^+^ cells | 7[2-48] | 14[8-31] | 0.35 |
| TIM3^+^ cells | 8[2-23.5] | 29.5[14.5-39.5] | 0.06 |
| PDL1^+^ cells | 27[0-74] | 11[2-25.5] | 0.35 |
| CTLA^+^ cells | 1[0-3] | 0.69[0-3.4] | 0.72 |

| **S4 Table**. HPV&HIV co-infection compared to HIV alone | | | |
| --- | --- | --- | --- |
|  | **HIV Only**  **(N=10)** | **HPV&HIV Co-infection**  **(N=19)** | **p Value** |
| Median Age (Years) | 58[54-60] | 55[47-63] | 0.64 |
| Smoking | 50% | 47.40% | 0.89 |
| Duration of lesion (months) | 11[8-12] | 10[5-24] | 0.73 |
| CD4 count (cells/µl) | 460[330-539] | 454.5[273-661] | 0.98 |
| Advanced stage disease | 0/9(0%) | 31.60% | 0.06 |
| p16 Positive | 9/9(100%) | 17/18(94.4%) | 0.47 |
| Abnormal p53 expression | 0/9(0%) | 1/17(5.9%) | 0.46 |
| CD3^+^ cells | 57[21-165] | 39[16-84] | 0.34 |
| CD8^+^ cells | 26[10-114] | 29[12-51] | 0.78 |
| CD103^+^ cells | 44[11-78] | 38[13-51] | 0.66 |
| FOXP3^+^ cells | 10[5-19] | 10[5-14] | 0.80 |
| CD68^+^ cells | 34[10-45] | 20[13-26] | 0.55 |
| CD163^+^ cells | 38[18-46] | 23[17-34] | 0.32 |
| LAG3^+^ cells | 26[1-50] | 17[10-28] | 0.72 |
| PD1^+^ cells | 9[1-48] | 14[8-31] | 0.46 |
| TIM3^+^ cells | 17[1-43] | 29.5[14.5-39.5] | 0.37 |
| PDL1^+^ cells | 6.5[0-33] | 11[2-25.5] | 0.65 |
| CTLA^+^ cells | 1[0.7-1.3] | 0.69[0-3.4] | 0.61 |

| **S5 Table.** Multiple hrHPV and penile squamous cell carcinoma | | | |
| --- | --- | --- | --- |
|  | **Single hrHPV**  **(N=9)** | **Multiple hrHPV (N=13)** | **p Value** |
| Median Age (Years) | 54[49-57] | 56[50-67] | 0.61 |
| Smoking | 55.60% | 38.50% | 0.43 |
| Duration of lesion (months) | 8[6-12] | 10[6-24] | 0.78 |
| HIV positive | 77.80% | 92.30% | 0.33 |
| HIV viral load (copies/ml) | 0[0-0] | 0[0-0] | 0.31 |
| CD4 count (cells/µl) | 575[445-737] | 356[245-729] | 0.26 |
| Advanced stage disease | 2/8(25%) | 38.50% | 0.53 |
| p16 Positive | 7/7(100%) | 92.30% | 0.45 |
| Abnormal p53 expression | 0/6(0%) | 7.70% | 0.49 |
| CD3^+^ cells | 26[8-64] | 39[32-54] | 0.24 |
| CD8^+^ cells | 24[8.5-42.5] | 34[12-44] | 0.44 |
| CD103^+^ cells | 22.5[4-40] | 38[20-51] | 0.72 |
| FOXP3^+^ cells | 10[9-14] | 10[4-12] | 0.45 |
| CD68^+^ cells | 16[13-21] | 25.5[17-30.5] | 0.06^*^ |
| CD163^+^ cells | 20[17-33] | 25.5[18-43] | 0.31 |
| LAG3^+^ cells | 11[6-17] | 20[12-30] | 0.15 |
| PD1^+^ cells | 41.5[17-60] | 13[7.5-21.5] | 0.13 |
| TIM3^+^ cells | 13[6-26] | 33[20.5-44] | **0.044** |
| PDL1^+^ cells | 19.5[0-27] | 11[3.5-36.5] | 0.77 |
| CTLA^+^ cells | 0.1[0-1.3] | 0.8[0-3.4] | 0.19 |
